# Autologous Platelet‐Rich Plasma and Shockwave Therapy for Refractory Middle Femoral Nonunion: A Case Report

**DOI:** 10.1002/ccr3.71400

**Published:** 2025-11-04

**Authors:** Chao Cheng, Ri‐chao Pang, Hua‐ge Wang, Zhong‐lin Xiao, Zheng‐dong Wang, Jin‐qi Zheng, Wenchun Wang

**Affiliations:** ^1^ Department of Rehabilitation Medicine People's Liberation Army the General Hospital of Western Theater Command Chengdu China; ^2^ Fourth Posting Clinic People's Liberation Army the General Hospital of Western Theater Command Chengdu China

**Keywords:** autologous platelet‐rich plasma therapy, case report, middle femoral nonunion, rehabilitation training, shockwave therapy

## Abstract

This case report details a patient with persistent middle femoral nonunion unresponsive to multiple surgeries. PRP therapy, shockwave intervention, and structured rehabilitation were combined to achieve clinical fracture healing. This multifaceted treatment approach illustrates the coordinated use of PRP and physical modalities, offering practical insight for the clinical treatment of complex bone nonunion.

## 1 Introduction

Fracture healing is a dynamic process that can be interrupted by adverse factors at any stage, leading to delayed or incomplete repair. The incidence of impaired fracture healing reaches approximately 8%–10% [1], while the nonunion rate for long bone fractures stands at about 5% [2]. When a fracture fails to heal within the anticipated timeframe, it is termed delayed union or nonunion. Although the definition of fracture nonunion remains under discussion, in China, the commonly accepted criteria define delayed union as failure to unite after 4 months, and nonunion as failure to unite after 8 months [3]. According to the FDA, fractures not healing within their expected period, typically 3–6 months, are considered delayed union, while a lack of healing over a 9‐month period with no radiographic progress for 3 consecutive months is labeled nonunion [4, 5]. The International Orthopedic Internal Fixation Association and the FDA both describe nonunion as the absence of healing within the expected clinical timeframe, with no signs of progress on imaging across a 3‐month span. The patient in this report experienced delayed healing of a mid‐femoral fracture after surgery, followed by a refracture, progressive bone resorption, and nonunion. Treating such cases poses significant challenges, and the likelihood of recovery is limited. However, after receiving platelet‐rich plasma therapy in conjunction with shockwave treatment and structured rehabilitation, the patient showed a favorable outcome. This case serves as a basis for sharing the diagnostic and therapeutic approach we adopted, in the hope that it offers practical value in managing similar cases.

## 2 Case Presentation

### 2.1 Case History and Physical Examination

**FIGURE 1:**
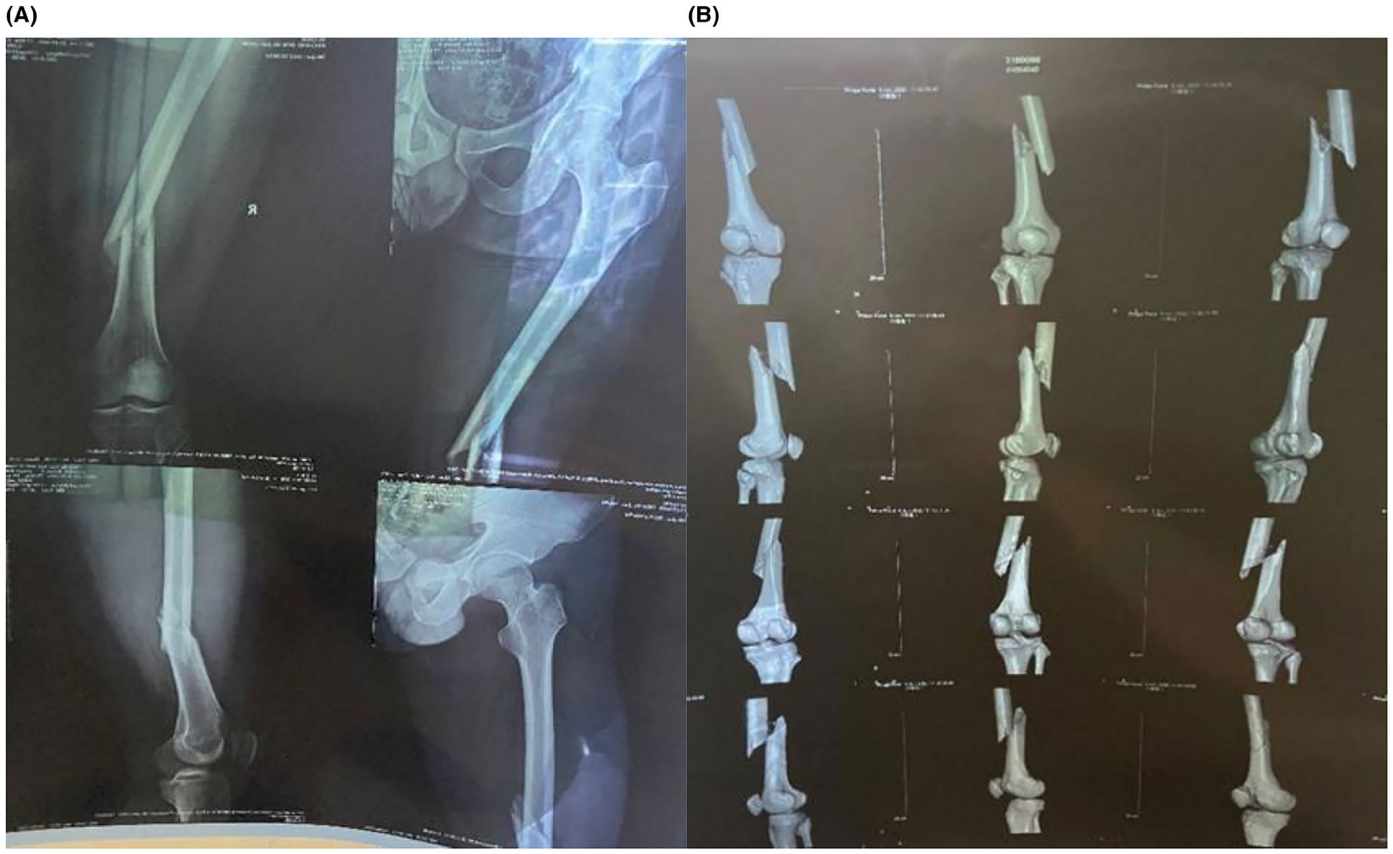
(A and B) X‐ray and CT imaging revealed a misaligned mid‐femoral fracture.

**FIGURE 2:**
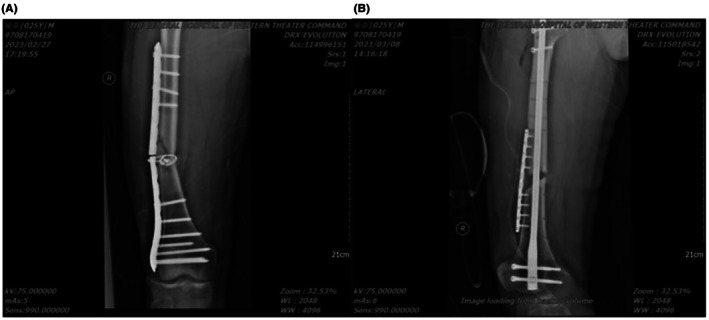
(A) X‐ray demonstrated failure of the internal fixation at the midsection, with visible bone discontinuity and angulation at the fracture site. (B) Follow‐up X‐ray 1 month postoperatively indicated persistent nonunion with signs of bone resorption.

A 26‐year‐old male presented with right thigh pain, limited mobility, and visible deformity after sustaining trauma from a heavy object (Figure 1A,B). Internal fixation using a plate and Kirschner nail was performed at a local hospital. Postoperative X‐rays confirmed proper alignment, with no signs of displacement or refracture (Figure 2A,B). Given the risk of delayed healing, conservative management was recommended. On February 27, 2023, while attempting to stand, the patient felt a sudden snap in the right thigh and lost the ability to move the limb. He immediately sought care at our hospital's Department of Orthopedics and underwent surgery on March 7, 2023. The procedure involved removal of the previous fixation, debridement and reduction of the nonunion site, iliac autograft harvesting, allograft placement, and internal fixation. Due to persistent nonunion, the patient was subsequently transferred to the Department of Rehabilitation Medicine for continued care.

On physical examination, the affected limb showed reduced circumference compared to the opposite side. The patient's VAS score while walking was 3. Limited ambulation ability, manifested as the need to walk with a single crutch and the distance being less than 500 m, although muscle strength and joint mobility remained within normal limits.

### 2.2 Imaging Examinations

## 3 Final Diagnosis

Middle femoral nonunion.

## 4 Treatment

The patient was transferred to the Department of Rehabilitation Medicine on March 24, 2023. Following clinical assessment and auxiliary investigations, a regimen combining autologous platelet‐rich plasma therapy with shockwave therapy was initiated on April 6, 2023.

Shockwave therapy was guided by X‐ray positioning. The targeted site was treated using a focusing head aligned along the determined line, with a pressure range of 2–4 bar and an impact frequency of 10 Hz. Each session involved 2000–3000 impacts, conducted once per week, over the course of five sessions. On each session of shockwave therapy, autologous platelet‐rich plasma treatment was also administered. The PRP was prepared using a blood component separator in the Department of Blood Transfusion, yielding pure platelet‐rich plasma (P‐PRP) with a concentration of (800–1000) × 10^9^/L, obtained through single blood cell collection. The collected PRP underwent quality control, then was stored at −80°C following freeze–thaw processing. Freeze‐thawed PRP (5 mL) was injected weekly, for a total of five injections per course.

**FIGURE 3:**
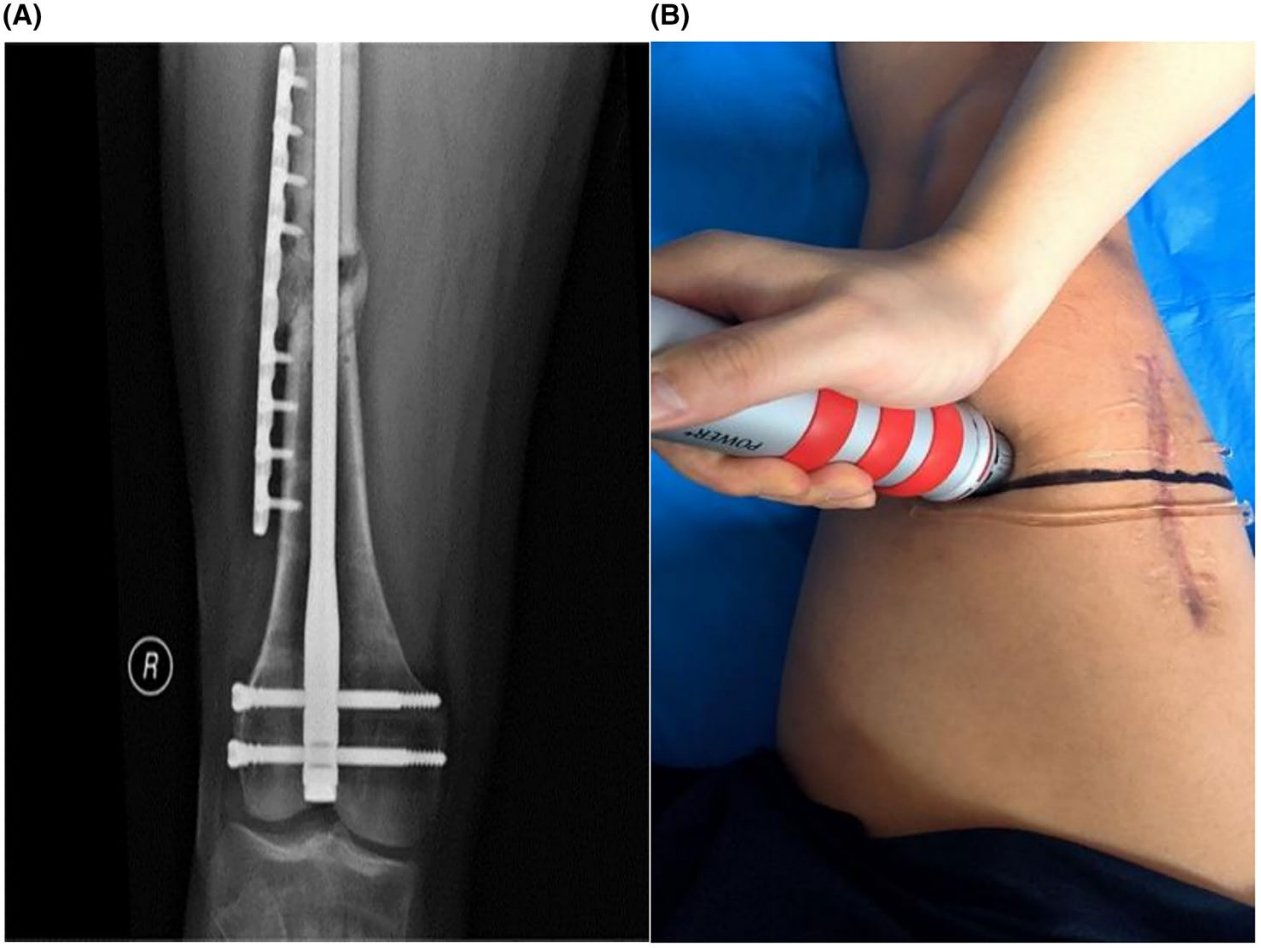
(A) A follow‐up X‐ray 1 month later showed partial callus formation. (B) The positioning approach was modified to ultrasound‐guided localization of all fracture ends for shockwave application.

A follow‐up X‐ray 1 month later (Figure 3A) showed partial callus formation. Given the presence of callus but persistent nonunion, a second round of combined autologous PRP and shockwave therapy was administered on July 27, 2023, with adjustments made to the treatment protocol.

**FIGURE 4:**
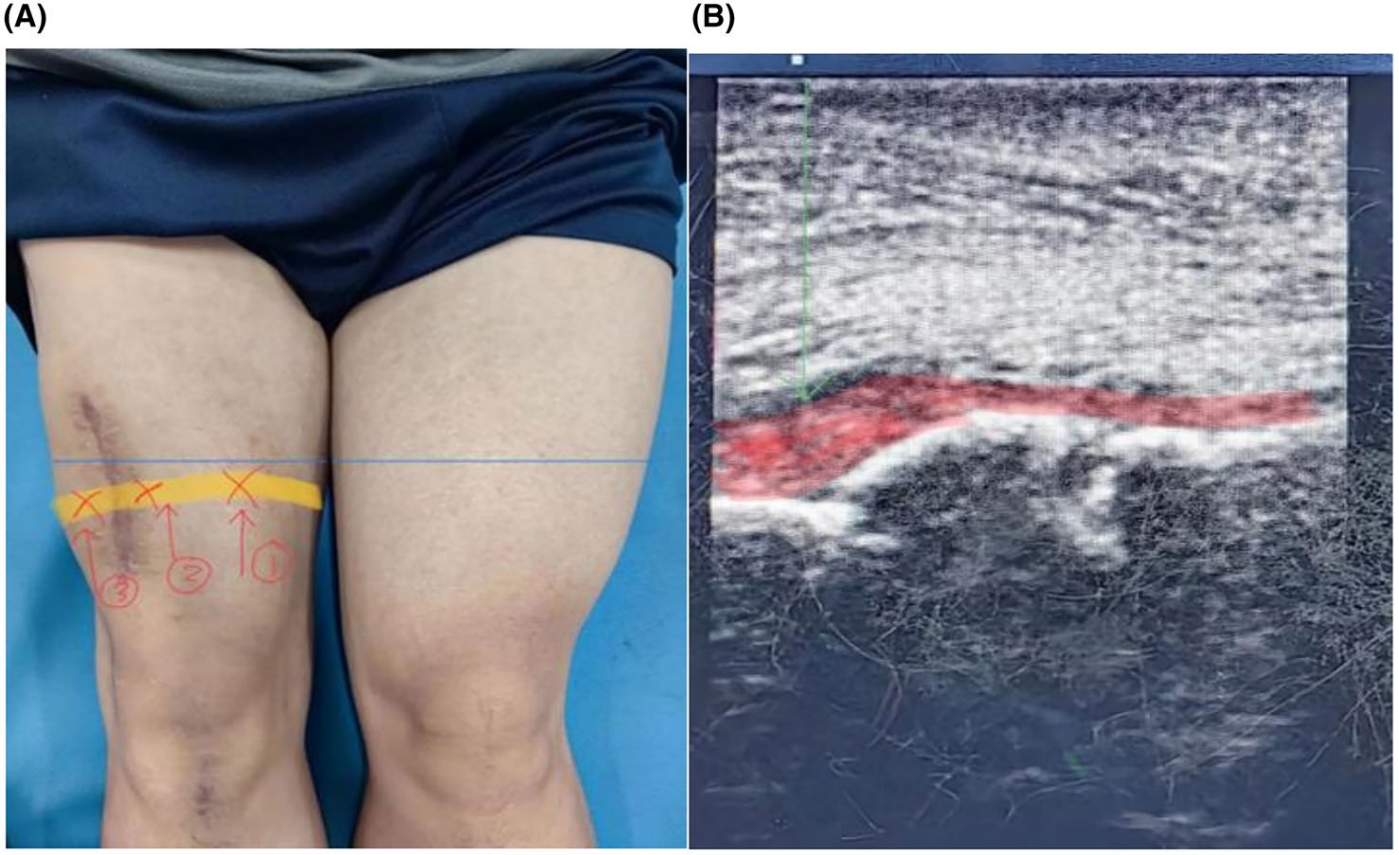
(A and B) Ultrasound‐guided multi‐point injection of PRP along localized markers for treatment.

The positioning approach was modified to ultrasound‐guided localization of all fracture ends for shockwave application (Figure 3B). For each session, 4–6 treatment points were selected along the target line using a focusing head. Pressure was maintained between 2 and 4 bar, with an impact frequency of 10 Hz. Each treatment point received 2000–3000 shocks. The therapy was administered once every other day, totaling five sessions. On each treatment day, autologous platelet‐rich plasma was injected at multiple sites under ultrasound guidance. A combination of freeze–thaw PRP and freshly prepared PRP was used weekly. Fresh PRP was prepared using a simplified device; after two rounds of centrifugation, 2 mL of plasma supernatant was obtained and combined with 10 mL of freeze–thawed PRP. A total of 12 mL of this mixed plasma suspension was injected into points ①, ②, and ③ (Figure 4A,B) once per week, for five sessions.

**FIGURE 5:**
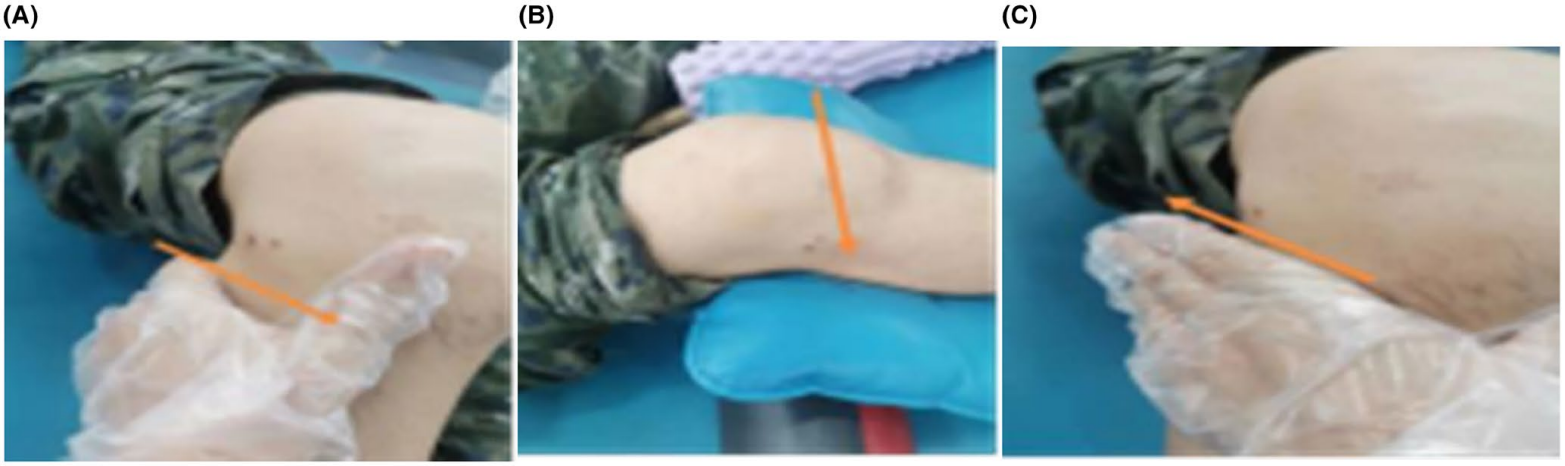
(A–C) To release tension in the mid‐quadriceps, lateral fascia, iliotibial band, hamstrings, popliteus, and popliteal fossa, joint mobilization techniques were applied to the tibiofemoral and patellofemoral joints.

To release tension in the mid‐quadriceps, lateral fascia, iliotibial band, hamstrings, popliteus, and popliteal fossa, joint mobilization techniques were applied to the tibiofemoral and patellofemoral joints (Figure 5A–C). Rehabilitation training focused on activating the medial head of the quadriceps and strengthening both the eccentric and concentric functions of the quadriceps and hamstrings. Pelvic stability exercises were also included. These exercises were performed in sets of 10 repetitions, 1–2 sets per day, 5 days per week.

## 5 Outcome and Follow‐Up

**FIGURE 6:**
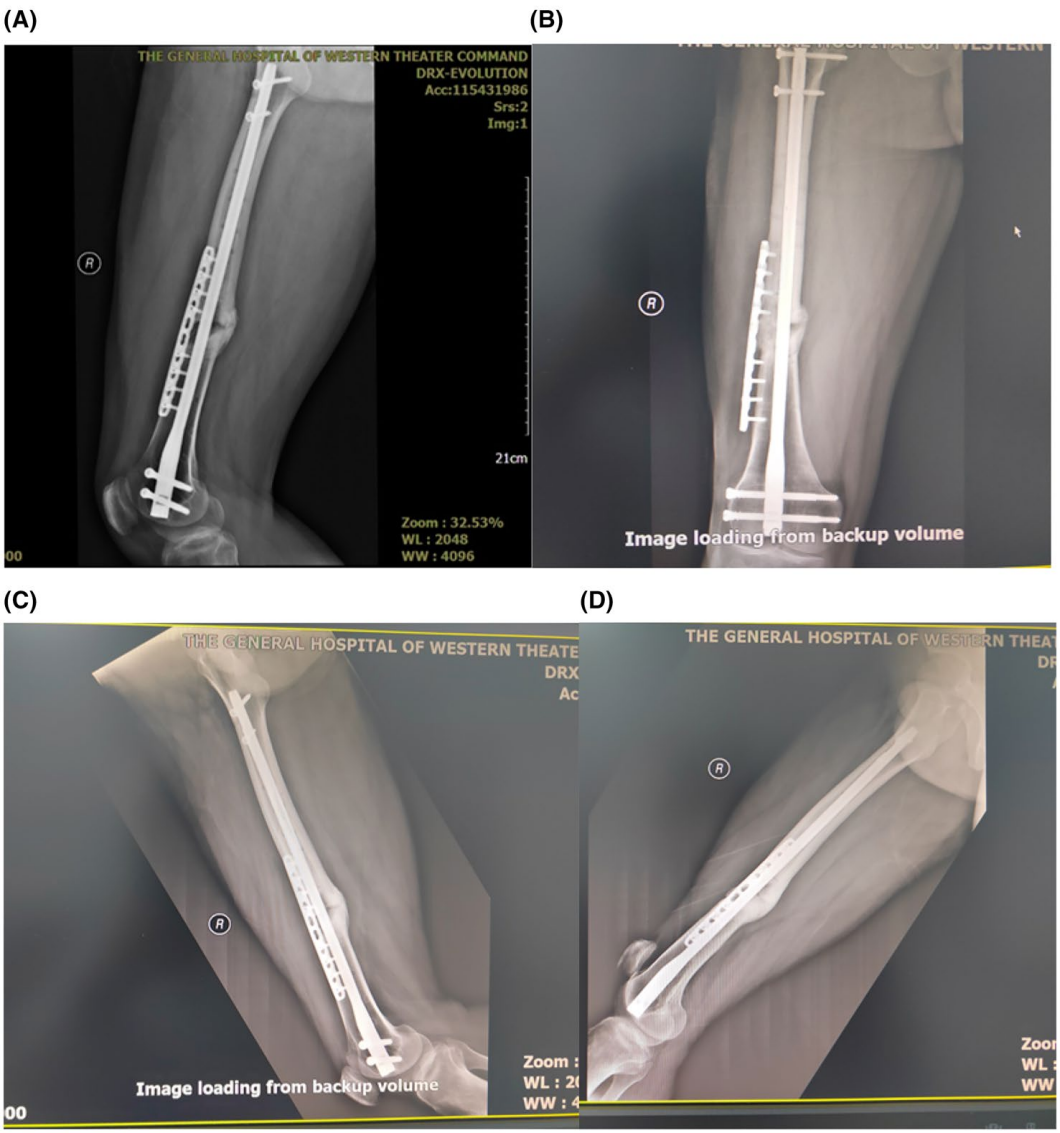
(A–D) Serial X‐ray evaluations were conducted over three consecutive months following rehabilitation, all of which showed clear callus formation.

The patient completed two full courses of autologous platelet‐rich plasma combined with shockwave therapy. Serial X‐ray evaluations were conducted over three consecutive months following rehabilitation, all of which showed clear callus formation (Figure 6A–D). After 3 months of follow‐up, the patient reported no pain while walking and was able to resume physical activities, including running distances up to 3000 m. Compared to his condition prior to treatment, both quality of life and capacity for social participation had significantly improved.

## 6 Discussion

Bone nonunion can arise from systemic conditions such as diabetes, metabolic disorders, and smoking, as well as local factors including infection and mechanical instability. Iatrogenic influences, severe trauma, suboptimal surgical intervention, and inappropriate postoperative loading, also contribute significantly to its development [6, 7].

Multiple strategies support bone healing, including bone induction, bone conduction, mechanical stabilization, and physical therapy modalities. Bone induction relies on the stimulation of undifferentiated pluripotent stem cells by tissues or biological extracts, triggering their differentiation into bone‐forming cells. Autologous platelet‐rich plasma (PRP) contains a range of cytokines such as BMP, VEGF, PDGF, TGF‐β1, IGF‐1, IL‐1β, and TNF‐α, all of which promote osteoblast differentiation and proliferation. This makes PRP a viable option for patients experiencing delayed union or nonunion [8, 9, 10, 11]. Extracorporeal shockwave therapy, a non‐invasive and safe intervention, operates primarily through the effects of pressure and cavitation at the nonunion site, creating microfractures that stimulate biological repair. This technique improves local microcirculation, triggers the release of growth factors, promotes cell activity, raises metabolic function, and promotes both inflammation and gene expression, while regulating signaling pathways involved in bone healing [12, 13]. For the patient in this case, the therapeutic protocol integrated two components: rehabilitation exercises and targeted mechanical stimulation. The aim was to regulate bone cell activity, reshape bone through adaptive mechanical stress, induce apoptosis in senescent bone cells, and maintain bone homeostasis [14]. Modifications to the therapeutic plan appeared to accelerate fracture healing relative to earlier phases. These improvements were likely related to the use of musculoskeletal ultrasound for precise shockwave positioning, and the application of ultrasound‐guided visual control during PRP administration [15]. The adoption of a multi‐point impact technique and refined injection strategy during the second round of shockwave therapy seemed to promote more effective callus formation compared to the previous single‐point approach. Additionally, the increased PRP dose allowed for deeper infiltration around the fracture site. Nonetheless, it remains unclear whether combining freeze–thaw PRP with fresh PRP alone significantly increases cytokine activity [16].

Our case report has several important limitations that must be considered. Firstly, although the case demonstrated a favorable outcome, as a single‐case report, it lacked a controlled study to establish a causal relationship between the combined treatment approach and the observed improvement, and therefore could only indicate a potential association. Secondly, the outcome assessment was primarily based on the patient's subjective report and clinical observations, which may introduce potential bias. Finally, the specific nature of this patient's disease course somewhat limits the generalisability of the findings to broader patient populations.

## 7 Conclusion

In summary, surgical management of femoral fractures should be integrated with mechanical principles to reduce the risk of instability and nonunion. Treatment strategies must emphasize fracture healing through standardized, multifaceted approaches and careful refinement of therapeutic protocols to maximize both healing outcomes and functional restoration. Although this case demonstrated favorable recovery, the theoretical foundation for the optimized treatment plan remains limited. We need to further investigate the underlying mechanisms and clinical randomized controlled studies and demonstrate the effectiveness of this approach with more clinical cases.

## Author Contributions

**Chao Cheng:** writing – original draft. **Ri‐chao Pang:** writing – review and editing. **Hua‐ge Wang:** resources. **Zhong‐lin Xiao:** validation. **Zheng‐dong Wang:** resources. **Jin‐qi Zheng:** validation. **Wenchun Wang:** writing – review and editing.

## Consent

The patient was provided the case report in advance of journal submission with ample time to provide feedback. The patient denied any additional commentary regarding patient perspective, and all patient feedback was incorporated into the case report prior to journal submission. After review of the case report, a consent form was reviewed with the patient, signed and is available on request.

## Conflicts of Interest

The authors declare no conflicts of interest.

## Data Availability

The data that support the findings of this study are available on request from the corresponding author. The data are not publicly available due to privacy or ethical restrictions.

## References

[1] G. M. Calori , M. Colombo , E. L. Mazza , et al., “Validation of the Non‐Union Scoring System in 300 Long Bone Non‐Unions,” Injury 45, no. 6 (2014): S93–S97, 10.1016/j.injury.2014.10.030. 25457326

[2] C. Tzioupis and P. V. Giannoudis , “Prevalence of Long‐Bone Non‐Unions,” Injury 38, no. 2 (2007): S3–S9, 10.1016/s0020-1383(07)80003-9. 17920415

[3] Z. Q. Lin , H. Z. Zhang , G. G. Luo , et al., “Comparison of 3 Treatment Methods for Distal Tibial Fractures: A Network Meta‐Analysis,” Medical Science Monitor 25 (2019): 7480–7487, 10.12659/MSM.917311. 31587012 PMC6792504

[4] J. W. Milgram , “Nonunion and Pseudarthrosis of Fracture Healing. A Histopathologic Study of 95 Human Specimens,” Clinical Orthopaedics and Related Research 268 (1991): 203–213. 2060209

[5] J. P. Frölke and P. Patka , “Definition and Classification of Fracture Non‐Unions,” Injury 38, no. 2 (2007): S19–S22, 10.1016/s0020-1383(07)80005-2. 17920413

[6] O. Ashman and A. M. Phillips , “Treatment of Non‐Unions With Bone Defects: Which Option and Why?,” Injury 44, no. 1 (2013): S43–S45, 10.1016/S0020-1383(13)70010-X. 23351870

[7] W. J. Metsemakers , N. Roels , A. Belmans , P. Reynders , and S. Nijs , “Risk Factors for Nonunion After Intramedullary Nailing of Femoral Shaft Fractures: Remaining Controversies,” Injury 46 (2015): 1601–1607, 10.1016/j.injury.2015.05.007. 26026201

[8] J. W. Belk , M. J. Kraeutler , D. A. Houck , et al., “Platelet‐Rich Plasma Versus Hyaluronic Acid for Knee Osteoarthritis: A Systematic Review and Meta‐Analysis of Randomized Controlled Trials,” American Journal of Sports Medicine 49, no. 1 (2021): 249–260. 32302218 10.1177/0363546520909397

[9] J. N. Katz , “Platelet‐Rich Plasma for Osteoarthritis and Achilles Tendinitis,” JAMA 326, no. 20 (2021): 2012. 34812886 10.1001/jama.2021.19540

[10] X. Li , X. Fu , Y. Gao , et al., “Expression of Tissue Inhibitor of Metalloproteinases‐1 and B‐Cell Lymphoma‐2 in the Synovial Membrane in Patients With Knee Osteoarthritis,” Experimental and Therapeutic Medicine 15 (2017): 885–889. 29399094 10.3892/etm.2017.5462 PMC5772747

[11] J. Fang , X. Wang , W. Jiang , et al., “Platelet‐Rich Plasma Therapy in the Treatment of Diseases Associated With Orthopedic Injuries,” Tissue Engineering Part B, Reviews 26, no. 6 (2020): 571–585. 32380937 10.1089/ten.teb.2019.0292 PMC9208862

[12] A. Willems , O. P. van der Jagt , and D. E. Meuffels , “Extracorporeal Shock Wave Treatment for Delayed Union and Nonunion Fractures: A Systematic Review,” Journal of Orthopaedic Trauma 33 (2019): 97–103, 10.1097/BOT.0000000000001361. 30570614

[13] N. Haffner , V. Antonic , D. Smolen , et al., “Extra‐Corporeal Shockwave Therapy(ESWT) Ameliorates Healing of Tibial Fracture Non‐Union Unresponsive to Conventional Therapy,” Injury 47 (2016): 1506–1513. 27158008 10.1016/j.injury.2016.04.010

[14] J. M. Hughes , C. M. Castellani , K. L. Popp , et al., “The Central Role of Osteocytes in the Four Adaptive Pathways of Bone's Mechanostat,” Exercise and Sport Sciences Reviews 48 (2020): 140–148, 10.1249/JES.0000000000000225. 32568926 PMC7310270

[15] T. Omodani , “Extracorporeal Shock Wave Therapy Combined With Platelet‐Rich Plasma Injection to Treat the Nonunion of a Stress Fracture of the Proximal Phalanx of the Great Toe: A Case Report,” Cureus 16, no. 3 (2024): e55877, 10.7759/cureus.55877. 38595890 PMC11002713

[16] T. Collins , D. Alexander , and B. Barkatali , “Platelet‐Rich Plasma: A Narrative Review,” EFORT Open Reviews 6 (2021): 225–235, 10.1302/2058-5241.6.200017. 34040800 PMC8142058

